# Liver transplantation in glycogen storage disease type III: A case‐series

**DOI:** 10.1002/jmd2.12463

**Published:** 2025-01-24

**Authors:** Simon Gay, Adrien Bigot, Louis d'Alteroche, Fanny Dujardin, Gaëlle Fromont‐Hankard, Nathalie Tressel, Ephrem Salame, François Maillot

**Affiliations:** ^1^ Internal Medicine Department Tours Hospital Tours France; ^2^ Tours University Tours France; ^3^ Hepatology Department Tours Hospital Tours France; ^4^ Pathological Department Pathological Department Tours Hospital Tours France; ^5^ Liver Transplant Department Tours Hospital Tours France; ^6^ UMR INSERM 1253, «iBraiN» Tours France

**Keywords:** case‐series, glycogen storage disease, liver, transplantation

## Abstract

Glycogen storage disease type III (GSD III) is a rare metabolic disorder characterized by a deficiency of liver and muscle amylo‐1,6‐glucosidase. This condition presents with severe hepatic symptoms in childhood, mostly hepatomegaly, hypoglycemia in half of patients, while muscular complications may predominate in adulthood. Hepatic fibrosis, cirrhosis and hepatocellular carcinoma (HCC) are common complications in older patients. Therefore, regular monitoring, including HCC screening, is essential for effective disease management. In some severe cases, liver transplantation (LT) may be necessary to treat life‐threatening complications. Here, we report the cases of three adult patients who required LT during the course of GSD III. Case #1: Diagnosis of GSD III was made in childhood, with development of hepatocellular carcinoma requiring partial hepatectomy followed by LT due to post‐operative complications. The patient recovered well and had favorable surveillance over a seven‐year period. Case #2: Diagnosis of GSD III in early childhood, with progression to cirrhosis in adulthood. Severe hepatic encephalopathy necessitated urgent transplantation, with a favorable recovery, although muscular symptoms remained present. Case #3: Diagnosis of GSD III in childhood, followed by later development of hepatocellular adenocarcinoma requiring LT. The patient recovered well and did not exhibit post‐transplant muscular symptoms. Post‐LT outcome was positive for all three GSD III patients, with significant improvement in liver function and no complications related to immunosuppression. Long‐term hepatic monitoring is essential for early detection of complications such as cirrhosis and HCC. LT indications should be individually evaluated, preferring less invasive options. These cases highlight the importance of a multidisciplinary approach to the effective management of GSD III, with particular attention to hepatic and muscular surveillance.

**Synopsis**

Here, we described 3 patients with GSD III, a rare inborn error metabolic disease, resulting in liver complications. We wanted to highlight the importance of liver screening and the possibility of liver transplant during the course of this disease.

## 1 INTRODUCTION

Glycogen storage disease type III (GSD III, OMIM 232400) is a rare autosomal recessive metabolic disease due to liver amylo‐1,6‐glucosidase (EC 3.2.1.33) deficiency (subtype IIIb), or liver and muscle deficiency (subtype IIIa) resulting from mutations in the *AGL* gene (1p21).^1^ It is characterized by both liver and muscle glycogen overload depending on genotypical variant. GSD III incidence is estimated at 1/83 000 in Europe and 1/100 000 in the USA.^2^ Some clusters exist within the Inuit population,^3^ Northern Africa Jewish patients,^4^ and in Faroe Islands.^5^ GSD III are divided into four subtypes, depending on which specific enzyme is affected in glycogen metabolism. GSD IIIa represents 85% of cases, in which clinical manifestations are hepatic and muscular. GSD IIIb occurs in 15% of cases and is primarily affecting the liver. GSD IIIc (glucosidase defect) affects only muscles and GSD IIId (transferase defect) affects muscle and liver tissues. GSD IIIc and IIId are extremely rare.^6^, ^7^, ^8^ Clinical symptoms are directly related to the accumulation of glycogen in the affected tissues. Early symptoms of hepatic damage are hepatomegaly and ascites. Liver failure is related to the accumulation of glycogen in the liver, which directly affects long term prognosis when cirrhosis appears. Hypoglycemia is a major symptom, resulting from the failure to release glucose in the blood. Muscle fatigue is directly related to the accumulation of intramuscular glycogen.^9^

Long term complications of GSD III include liver, muscle and heart issues. As such, adult patients with GSD III may develop liver fibrosis, leading to cirrhosis and hepatocellular carcinoma (HCC). Incidence of malignant transformation of HA to HCC is rare, estimated at 15%,^10^ but HCC may also develop without any pre‐existing adenomas, and without any evidence of liver cirrhosis.^11^, ^12^ Incidence of cirrhosis in GSD III is variable, from 10% to 40%, with a median age of 40 years at diagnosis.^9^, ^10^, ^13^ Hepatic complications screening is mainly based on annual abdominal ultrasounds. In adults, annual hepatic MRI with gadolinium contrast is recommended because of its better diagnostic performances for small hepatic lesions.^14^

In our metabolic center dedicated to adults with inherited metabolic disease, we used to screen for cirrhosis and HCC in patients with GSDIII. Hence, we used to prescribe for 20 years a yearly liver imaging (systematic US scan and MRI on demand) as well as plasma liver tests and alphafoeto protein. The systematic follow‐up has identified liver complications in three patients, all leading to liver transplantation (LT). Thus, we describe cases of three GSD III patients who benefited from a LT.

## 2 CASE REPORTS

### 2.1 #Case 1

Patient 1, born in 1952, has been diagnosed with GSD III during childhood after hypoglycemia. Diagnosis was biologically confirmed in 2000 by a low amylo‐1,6‐glucosidase activity (−0.18 nmol glucose/min/mg vs. control) in leukocytes associated with an increase in the amount of glycogen in erythrocytes (486 μg/g, normal <75 μg/g). He was used to eat cornstarch before physical effort since childhood.

In 2014, the patient was admitted for dyspnea as part of a restrictive respiratory failure related with a left diaphragmatic paralysis. At that time, the left diaphragmatic paralysis was related with muscular involvement in GSD and specific respiratory treatment were started. Routine screening for HCC revealed an increase in alpha foeto protein (79 IU, normal <30 IU). US scan showed a 16 × 11 mm pseudonodular formation at the expense of the hepatic IV segment, confirmed by MRI. Research for extrahepatic disseminating tumor using thoraco‐abdomino‐pelvic CT scan was negative. The patient had a partial hepatectomy including segment IV and VIII in March 2015. Pathological examination of the hepatectomy showed three foci of HCC, from 1.5 to 2.2 cm. Analysis of non‐tumoral liver tissue confirmed the diagnosis of an early‐stage cirrhosis with septal fibrosis. Following liver surgery, the evolution was characterized by bleeding and early infection at the surgical site requiring broad‐spectrum antibiotic therapy for 14 days. After several local complications over a month (biliary anastomoses, installation of biliary prosthesis, periprosthetic hepatic abscesses) the patient was finally listed as a LT emergency, secondarily to major liver failure with encephalopathy and jaundice. He was then transplanted on 27/07/2015. Pathological analysis of the explanted liver showed cirrhosis with cytologic patterns related with glycogenosis, with some nodules in low‐grade dysplasia, but especially large ranges of ischemic necrosis. The aftermath was marked by septic shock in intensive care. Four days later, and after appropriate care, the patient recovered normal liver function. Surveillance of the immunosuppressant graft was good over time, with no local complications on CT reassessments. His muscular symptoms and dyspnea improved following LT and functional rehabilitation. The patient indicated no need for specific regimen, since he experienced an improvement of muscle strength over time and no longer need for non‐invasive ventilation.

### 2.2 #Case 2

**FIGURE 1:**
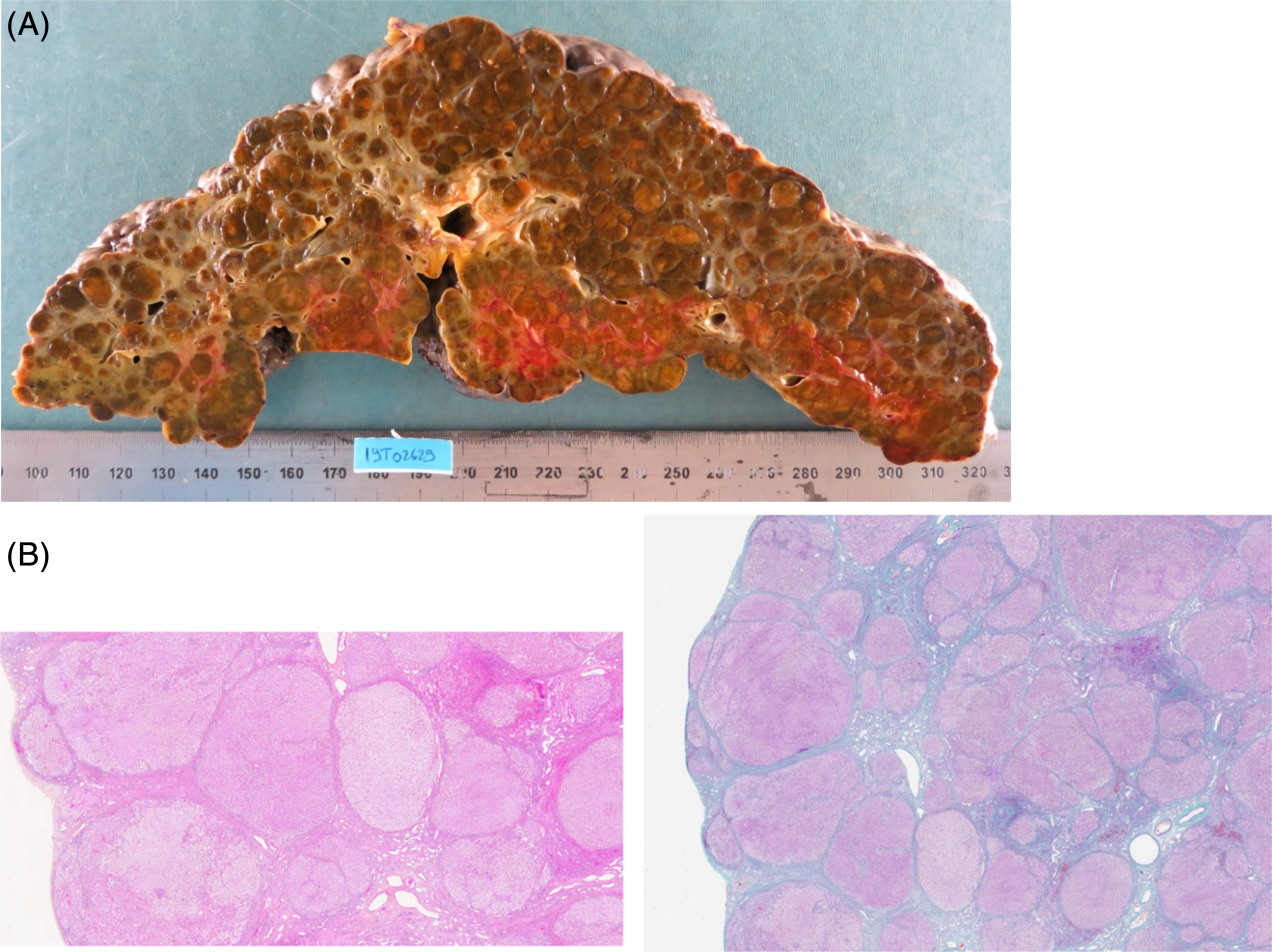
Macroscopic and pathological finding of patient #2. (A) Macroscopic aspect of the explant with macronodular cirrhosis. (B) Pathological staining of liver explant. Left: Hematoxylin‐ephloxine‐safran coloration on liver tissue, with cirrhotic aspect (nodulisation of liver tissue). Right Masson Trichrome coloration on liver tissue, with mutilating fibrosis (green).

Patient 2, born in 1975, has been diagnosed with GSD III at the age of 10 months after severe hypoglycemia. Diagnosis was performed in 1976 by showing a decrease in amylo‐1,6‐glucosidase activity on a liver biopsy with an accumulation of glycogen in hepatocytes. No molecular analysis was performed. At the age of 19, the diagnosis of partial epilepsy was made, treated with LAMOTRIGINE 300 mg per day. The patient experienced several episodes of hypoglycemia, often leading to seizures. She seemed to be observant, especially with her regimen (high protein intake >30% of total alimentation) and cornstarch intake before physical activity. Liver function monitoring detected cirrhosis at the age of 20 years, initially with no signs of HCC. Esophageal varices were treated endoscopically in 2003. Subsequent monitoring from 2004 to 2019 revealed no liver tests abnormalities, with subtle symptoms of liver failure or portal hypertension. At the age of 44 years, the patient's condition deteriorated due to dermo‐hypodermitis of the right lower limb, accompanied by hepatic encephalopathy. Given the rapid decline in liver function (quick time 13%, bilirubinemia at 708 μM/L) and a dire short‐term prognosis, the patient was promptly placed on transplant list on 08/3/2019. She underwent an emergency LT on 19/03/19. In perioperative term, she benefited from vasopressive treatment and fluid expansion. High energetic and protein intakes were introduced after metabolical and infectious adverse events abated. Pathological of explanted liver (Figure 1) showed cirrhosis with dysplastic nodules, one HCC foci of 0.7 cm. Hepatic encephalopathy, cytolysis and cholestasis regressed in the post‐operative period. At long term follow‐up, the patient continued to experience muscular fatigue limiting physical activity. Morphological and function monitoring of the transplanted liver showed satisfactory outcomes. At last show up in 2024, liver function was normal, and the patient had no further hypoglycemia. Yet, muscle impairment was persistent without evidence of any acute rhabdomyolysis episode. High protein regimen was maintained.

### 2.3 #Case 3

**FIGURE 2:**
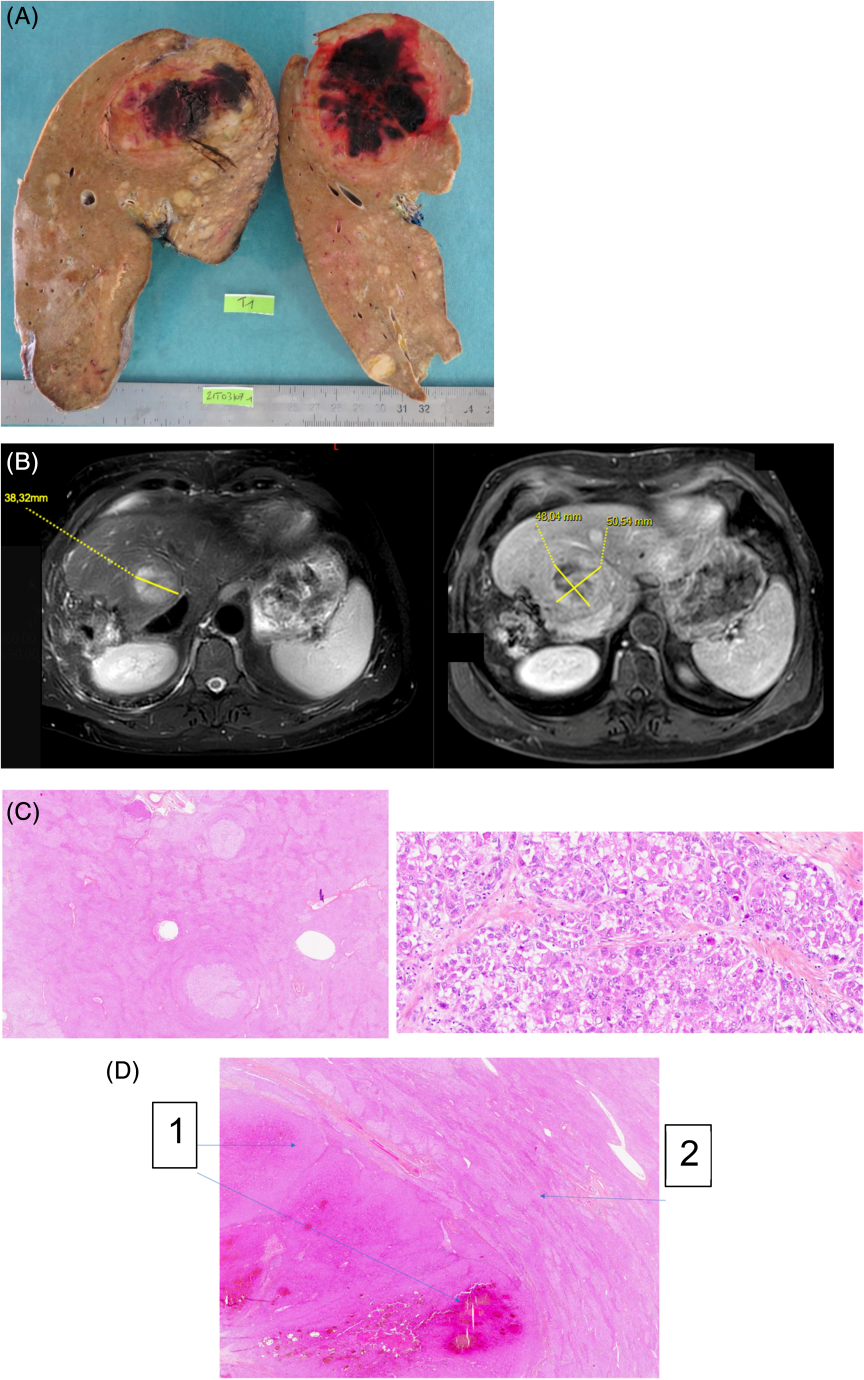
Macroscopic, imaging and pathological findings of patient #3. (A) Macroscopic aspect of the explant with voluminous liver adenoma, HCC nodules without cirrhosis. (B) Liver MRI. Left: T1 MRI with gadolinium enhancement. Right: T2 MRI. (C) Pathological exam on liver tissue, HES staining. Left: Heterogenous mosaic glycogen overload related with glycogenosis. Right: HCC with cytonuclear modification and architectural modification. (D) HES staining. (1) Liver adenoma with hemorrhagic transformation. (2) Normal liver tissue.

Patient 3, born in 1970, has been diagnosed with GSD III at the age of 1 year after severe hypoglycemia and benefited from a porto‐cave derivation at the age of 3 years. GSD III has been confirmed further by molecular analysis showing mutations (c18_19del and c.26 81+1G>A) in the *AGL* gene. The patient seemed to be lost to follow‐up during early adulthood. No specific regimen was taken. The patient never reported any muscle symptoms. In 2015, systematic screening by US scan and liver MRI showed a liver tumor (Figure 2) leading to right hepatectomy in 2016. Whole body imaging with PET scan was performed to exclude metastasis at that time. Pathological analysis (Figure 2) showed glycogen accumulation related with GSD and a moderately‐differentiated HCC of 10 cm as well an unclassified adenoma of 11 cm, without any evidence of cirrhosis. The size of HCC was probably favored by porto‐caval shunt since the age of three. The patient was placed on the transplant list from that moment on, to prevent further degeneration of the remaining liver. Surveillance between 2016 and 2021 showed a progressive increased size of hepatic lesions corresponding to adenomas. The presence of a suspicious tumor, tripling in volume in 4 years led to LT on 07/04/21. High protein and glucose intake was permitted with intravenous fluids during perioperative term. Pathological analysis of the liver showed a voluminous unclassified hepatocellular adenoma of 7.7 cm as well as two well differentiated HCC of 3.1 and 2 cm. Non tumoral liver tissue was characterized by portal and septa fibrosis without constituted cirrhosis. Infectious complications occurred on surgical site during post‐op period, amended under antibiotics well conducted. The patient's liver function recovered and no longer showed signs of cirrhosis. At last show up in 2023, the patient related no adverse event and had a normal life under immunosuppressive treatment. No specific dietary regimen was maintained (Table 1).

**TABLE 1:** Patients characteristics.

	Clinical presentation at diagnosis	Screening	Biochemical diagnosis	Genotype	LT indication	Perioperative period^a^, ^b^	Histological findings (surgery and explant)
#1	Hypoglycemia Muscle impairment	HCC without cirrhosis	Amylo‐1,6‐glucosidase activity low in leucocytes (−0.18 compared with standard) with increase of glycogen in erythrocytes (486 μg/g)	N.A.	HCC lesions and post‐hepatectomy complications	559 UI/L/3.7 mmol/L^a^ Polyionic glucose fluid 5% 1.5 L/day^b^ Parenteral nutrition introduced at 10 days post‐LT	Cirrhosis 3 HCC
#2	Hypoglycemia Early cirrhosis with specific complications	Chronic liver failure Portal hypertension Esophageal varices	Liver biopsy in 1976: decreasing amylo‐1,6‐glucosidase activity (0 HERS/g) with increase of glycogen in hepatocytes (13.7 g/100 g liver tissue)	N.A.	Acute liver failure after sepsis	2388 UI/L/3.5 mmol/L^a^ Intravenous glucose fluid 10% 1.5 L/day^b^ Parenteral nutrition introduced 14 days post‐LT	Cirrhosis with multiple dysplastic nodules. Among them, one HCC foci (0.7 cm)
#3	Hypoglycemia	Low impact on quality of life, only specified regime	N.A.	AGL gene (c18_19del and c.26 81+1G>A)	Increasing tumoral lesion while screening	1680 UI/L/2.3 mmol/L^a^ Polyionic glucose fluid 5% 1.5 L/day^b^ Oral nutrition was authorized at 3 days after LT	Moderately differentiated HCC (10 cm) and unclassified adenoma (11 cm) Fibrosis without cirrhosis Explant: Unclassified HA (7.7 cm) and 2 HCC (3.1 and 2 cm)

*Note*: Clinical characteristics of our patients including predominant symptoms, clinical follow up data, biochemical and genotype diagnosis, LT indication and pathological patterns.

^a^ Data including CK (in UI/L)/lactate (in mmol/L) when available.

^b^ Protocol for glucose infusion and protein intake during LT.

## 3 DISCUSSION

The present report describes the clinical course of three patients affected by GSD III, before and after LT. LT indications were markedly different between these three patients. As such, Case #2 was transplanted for an end‐stage cirrhosis, Case #3 for rapidly growing HCC and Case #1 because of post‐operative complications of liver surgery for localized HCC. Such cases illustrate the need to individualize the indications of LT. Indeed, LT is the ultimate procedure, mostly proposed in end‐stage liver disease, rapidly growing tumors (Case #3) or life‐threatening complications in cirrhotic state (Case #2).^14^ Although, LT indications were different, it is of note to underline an overall favorable post‐LT outcome in our three patients, as we observed significant improvement in liver function and no complications related to immunodepression.

Our case‐series illustrates that LT did not correct the underlying amylo‐1,6‐glucosidase muscular deficit associated with GSD III, explaining the persistence of exercise intolerance after LT, especially in patient 2. On the opposite, LT has suppressed the risk of hypoglycemia. Most reported cases have occurred in infants with inadequate metabolic control,^15^, ^16^ yielding significantly positive outcomes in terms of alleviating severe hypoglycemia. Concerning specific regimen after LT, it seems indicated to pursue high protein intake and preventive meals before physical activity depending on the intensity of muscle impairment. With the multiplication of liver transplant centers, and improvement in life expectancy in inherited metabolic diseases, the criteria for transplant candidacy have expanded, particularly in young adults. Predominant indications now include the progression of cirrhosis and associated complications.^17^

French guidelines for GSD III management insists on strict hepatic monitoring.^14^ In children, it is recommended to perform liver US scan annually. In adults, an annual hepatic MRI with contrasts agents such as gadolinium seems to be effective to detect intrahepatic nodules and indirect signs of cirrhosis. Such monitoring has to be conducted on a long term basis for early detection of cirrhosis and HCC at early stage.^13^ As there is no evidence‐based strategy to reduce the risks for liver complications in GSD III, close hepatic monitoring is mandatory.

Elastometry techniques are not validated for GSD III but can help clinicians detecting fibrosis. Liver biopsies seem to be useful in rapidly growing lesions or changing appearances on imaging but are not essential to diagnose cirrhosis. In the patients we report here, pathological patterns including inflammatory or unclassified adenomas are related with increasing risk of malignant transformation.

The question of liver transplant in GSD III is multifaceted within the literature. Considering the incidence of early hepatic complications, typically in the third or fourth decade of life, along with favorable outcomes related to hypoglycemia risk reduction and oncologic management; advocating for LT is GSD III appears reasonable in specific circumstances. Moreover, postoperative data offer reassurance, without any reported recurrence of HCC, normalization of liver function and overall favorable health outcomes following LT, as illustrated in our case‐series.

## ETHICS STATEMENT

Ethics statement are provided in supporting information.

## Supporting information


**Appendix S1:** Supporting information.

## Data Availability

All data are available on request, contacting the main author.
