# Supplementary material for: Liver transplantation in glycogen storage disease type III: A case‐series
Source: JIMD Rep. 2025 Jan 24;66(1):e12463. doi: 10.1002/jmd2.12463 (PMC11758152; doi:10.1002/jmd2.12463)

Compliance with Ethics Guidelines

Simon Gay, François Maillot, Nathalie Tressel, Ephrem Salame, Louis D'alteroche, Fanny Dujardin, Gaelle Fromont Hankard declare that they have no conflict of interest.

Informed Consent
All procedures followed were in accordance with the ethical standards of the responsible committee on human experimentation (institutional and national) and with the Helsinki Declaration of 1975, as revised in 2000 (5). Informed consent was obtained from all patients for being included in the study.
Proof that informed consent was obtained must be available upon request

Additional informed consent was obtained from all patients for which identifying information is included in this article.

This article does not contain any studies with animal subjects performed by the any of the authors.

Details of the contributions of individual authors

Simon Gay and François Maillot were the main authors.

Fanny Dujardin helping reviewing pathological data from liver transplant.

Louis D'Alteroche & Ephrem Salame contributed to liver screening and cirrhosis complication management with patients.

Simon GAY 30/10/2024

Main author

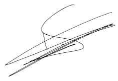

Supplement: Supplementary file 1 — Appendix S1: Supporting information. [file JMD2-66-e12463-s001.pdf]
